# Pathologic dislocation of the shoulder secondary to septic arthritis: a case report

**DOI:** 10.1186/1757-1626-2-9131

**Published:** 2009-12-02

**Authors:** Farshid Bagheri, Mohamad H Ebrahimzadeh, Seyed Reza Sharifi, Hossien Ahmadzadeh-Chabok, Javad Khajah-Mozaffari, Asieh S Fattahi

**Affiliations:** 1 Orthopedic Research Center, Ghaem Hospital, Mashad University of Medical Sciences, Mashad 91766-99199, Iran

## Abstract

Septic arthritis of the shoulder is uncommon in adults, and complete dislocation of the glenohumeral joint following septic arthritis is extremely rare. We report a case of pathologic shoulder dislocation secondary to septic arthritis in an intravenous drug abuser.

## Background

Septic arthritis of shoulder is an uncommon problem. Non-traumatic shoulder subluxation resulting from hemiplegia or brachial plexus injury has been reported [1]. There are few reports of glenohumeral subluxation following septic arthritis [2,3]. Based on the authors' knowledge, there is no report of complete dislocation of the glenohumeral joint secondary to shoulder joint infection in intravenous - drug abusers in the English medical literature.

## Case presentation

A 32-year-old man with a 2 weeks history of left shoulder pain and limited range of motion was referred to our orthopedic clinic at Ghaem Hospital, Mashad University of Medical Science, Iran. He complained of severe pain and restricted ROM of his non-dominant left shoulder. There was no history of direct or indirect trauma to the left shoulder but he reported frequent intravenous heroin injection on his left upper limb. On physical examination, the patient was afebrile, but warmness, swelling and tenderness of the left shoulder were noted. Range of motion was limited in all directions and the humeral head was palpable in antromedial of the shoulder area.

**Figure 1:**
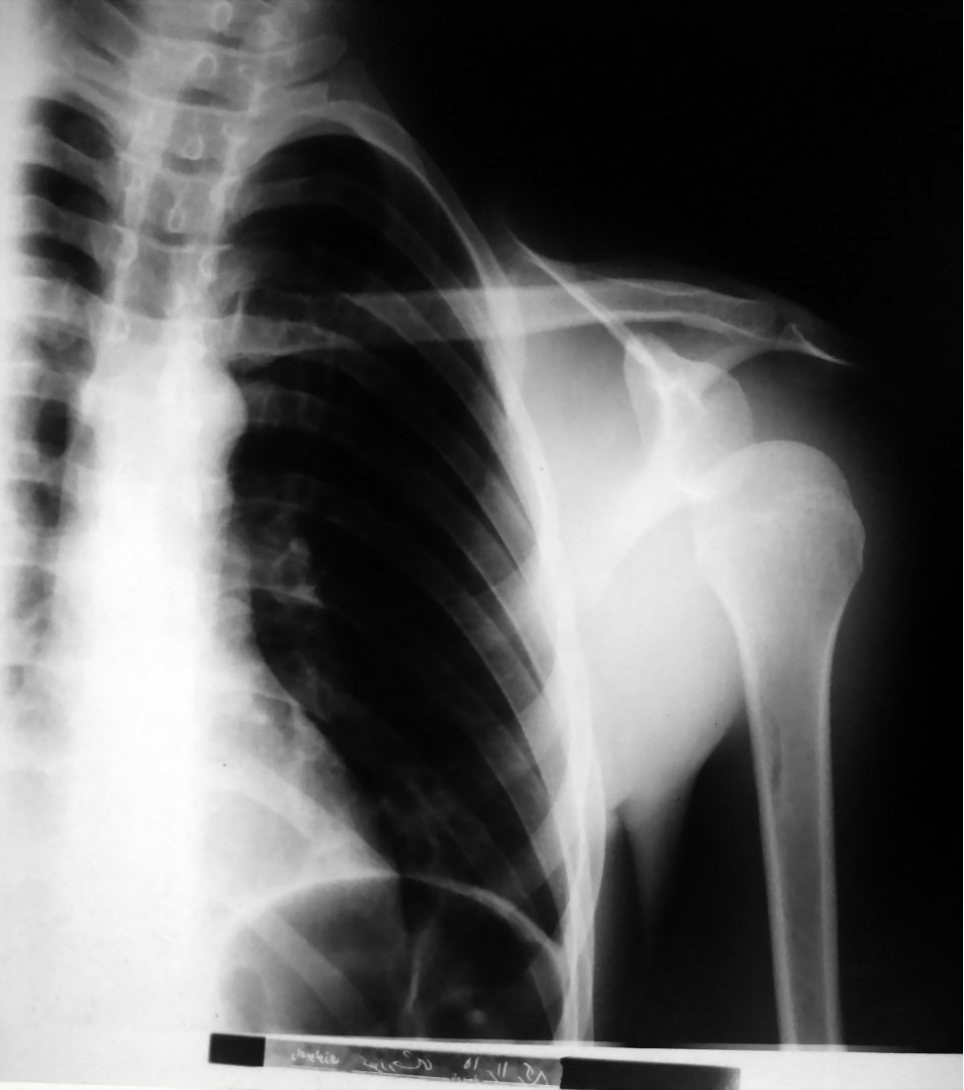
**AP x-ray of the left shoulder shows anteroinferior dislocation**.

On radiography a complete anteromedial glenohumeral dislocation was evident (Fig 1). The patient was admitted to our orthopedic department. The laboratory examination reported an elevated ESR (ESR = 50), positive CRP and leukocytosis (WBC = 14000 with 80% PMN). Glenohumeral joint puncture revealed obvious dark creamy pus. The patient was taken to the operating room, and with the diagnosis of shoulder septic arthritis and secondary shoulder dislocation, he underwent an open arthrotomy via a deltopectoral approach. While we incised the skin and subcutaneous tissue, a lot of puss drained out from rotator interval. About 350 cc of cloudy brown fluid were drained out from the joint and subacromial bursa. Fluid culture and tissue specimen was taken. Massive granulation tissue and necrotic tissue could be seen throughout the joint and adjacent bursa. We attempted through debridement and irrigated the joint with 10 liters of sterile normal saline. Rotator cuff muscles and long head of biceps appeared intact. Anterior and inferiorly dislocated humeral head spontaneously reduced. We did not suture synovium and rotator interval. Soft tissue was closed over suction drain and Velpeau bandage was applied on the left upper limb, which continued for 2 weeks. After surgical drainage the patient felt better and his general condition improved. Tenderness and pain in affected joint decreased remarkably.

**Figure 2:**
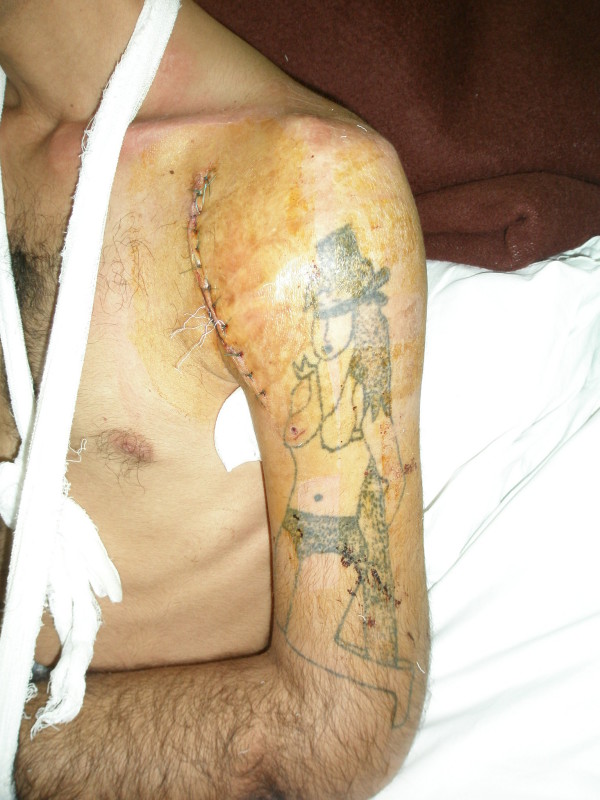
**A photo of the patient postoperative**.

**Figure 3:**
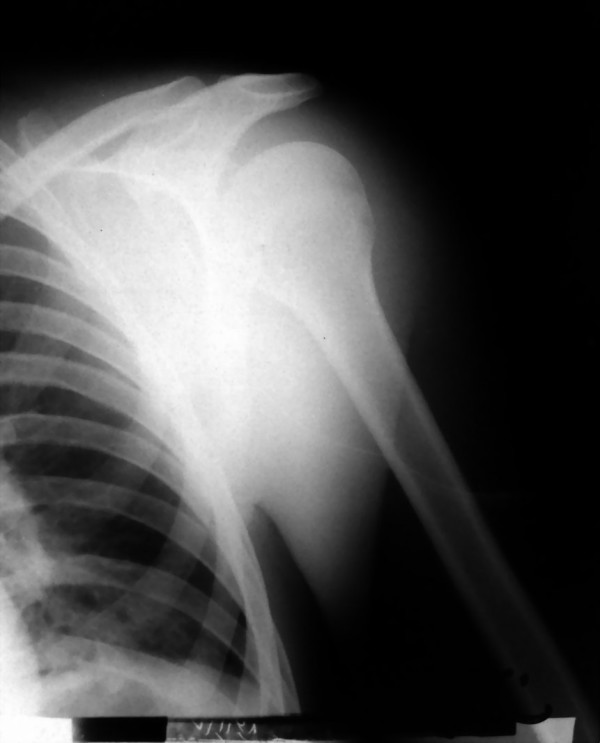
**Post-operative x-ray shows reduction**.

Cultures identified methicilin-resistant staphylococcus areus. We administered intravenous vancomycin and clindamycin based on microbial sensitivity results. After 2 weeks of intravenous antibiotic therapy, patient's general condition greatly improved and laboratory markers of infection (ESR, CRP, CBC) decreased. He was discharged at 2 weeks post operation with oral antibiotic (cloxascillin and clindamycin) for the next 4 weeks. The limb was simply immobilized in a sling. (Fig 2) and pendulum exercises and passive range of motion exercises was advised. We visited the patient at 4 weeks postoperation. He was satisfied with decreased pain and the sense of well being. We discontinued sling and immobilization and prescribed progressive strengthening exercises. Follow-up x-ray at 4 weeks post operation revealed acceptable reduction with no sign of subluxation (Fig 3). Also, we could not detect any sign of avascular necrosis of humeral head in control radiographies. Range of motion was almost normal and there was no Sign of AVN, subluxation or generation at 12 months post operation visit.

The authors have got informed consent forms signed by the patient for both print and electronic publication of the case and all its components; photographs, images and x-rays.

## Discussion and Conclusion

The most common cause of septic arthritis is hematogenous but recently, the incidence of hematogenous septic arthritis has declined significantly, while local injection-induced septic arthritic has been increased specially in shoulder. Intravenous drug abusers are at great risk of septic arthritis of unusual joints with unusual organisms [4].

Septic arthritis of the glenohumeral joint is relatively rare compared with knee and hip joints, thus the diagnosis requires a high index of suspicion and early evaluation of the affected shoulder by the clinician. In a review of English literature from 1960 to 1996 by Lossos 167 cases of shoulder septic arthritis was reported [1,5,6] However, the incidence of septic arthritis of the shoulder may be increasing. This may be associated with aging of the population, the increased survival rate of elderly patients who have chronic debilitating diseases and the increase of intravenous drug abuse and HIV positive patients [7-10].

A few reports of septic arthritis causing shoulder subluxation have appeared in the literature. Gompris and Darlington described a case of rheumatoid arthritis and shoulder septic arthritis causing bilateral glonohumeral dislocation [2].

Gordon and Hatchful reported a case of inferior shoulder displacement following pyarthrosis [4].

Ranking K and Rycken reported on an adolescent with bilateral dislocation of proximal humeral epiphyses [11,12].

There are also three reported cases of drooping of the shoulder because of septic arthritis [5,7,2]. The present case happened in an intravenous-drug abuser, who tried to hide his addiction. The volume of fluid/pus necessary to cause a dislocation in glonohumeral joint is not known [3].

The exact mechanism of shoulder dislocation in septic glenohumeral arthritis has not verified yet.

Presumable mechanism may be incompetency of glenohumeral ligaments due to gradual expansion of joint capsule as the fluid/pus accumulates slowly. Expanded loose glenohumeral capsule and its intimately associated ligaments (glenohumeral ligaments) result in incompetency of major static stabilizers of the inherently unstable shoulder joint. Gradual accumulation of fluid/pus seems to be essential prerequisite for the dislocation to occur.

Sudden expansion of joint capsule produces intolerable pain and the patients seeks treatment as soon as possible and dislocation can not be expected.

If the patient is somehow insensitive to pain or intentionally suppresses the pain with narcotics (intravenous drug abusers), fluid/pus can accumulates in the joint in large volumes, distending joint capsule and ligaments gradually and finally result in dislocation. It needs further studies to specify whether these pathologic changes would lead in recurrent dislocation.

Orthopedic surgeons should be familiar with this uncommon complication of shoulder septic arthritis particularly in patients who are intravenous-drug abusers and have immunodeficiency conditions. Intravenous drug abuse, itself, can be considered a risk factor for articular infection [13]. Ang-fonte et al attribute a fourfold increase of septic arthritis to this factor [6].

Furthermore, the shoulder is the less commonly involved joint among drug abusers [3]. But we should have high suspicion of this disease especially in immune compromised and intravenous drug abuser patients.

## Consent

The patient has signed out a formal consent form for publication of this case and pictures.

## Competing interests

The authors declare that they have no competing interests.

## Authors' contributions

FB, MHE, SRS, HAC, MKM admitted the patient and performed surgery, MHE, HAC, MKM and ASF followed the patient and contributed in writing the MS. All authors read the final manuscript and approved the text.
